# Colica Pictonum

**Published:** 1829

**Authors:** 


31. *Colica Pictonum.*—In the Journal of Foreign Medicine, for June, 1829, we find an account of a paper on this distressing and obstinate disease, by Dr. Montanciex. It consists of a series of cases treated in the hospital of St. Antoine, by Dr. Kapelar, with Alum, (*sulphas aluminæ et potassæ.*) This, as our readers are aware, is an old remedy; but it was formerly given in small doses, compared with those employed by Dr. K. Passing over the narrative of cases, we shall transcribe the general observations at the close of the article, in the hope that some of our friends may be induced to give the practice of St. Antoine a full and fair trial.

Thirteen years' experience has proved the value of the proposed remedy, which is both innocent and effectual.

Neither inflammation of the stomach or bowels has ever occurred. In most cases, three or four drachms of the alum were sufficient to render the patient convalescent, and in no one instance has a relapse happened. M. Montanciex has seen the patients frequently after their discharge, and is therefore able to speak confidently upon this point.

It will be perceived that the requisite dose of the remedy is not always in proportion to the severity of the disease. Cases, which commenced with very alarming symptoms, yielded to two or three drachms; others, which appeared mild in their character, resisted even eight or ten drachms. The physician must therefore act according to circumstances, but the first dose should not be more than one drachm.

The sulphate of alum is asserted to be the best remedy we possess against the metallic colic. We think, observes M. Montanciex, that it will entirely supersede every other medicine, if its effects are impartially compared with other modes of cure.

It is well known, that Colica Pictonum is often followed by paralytic affections of particular muscles, especially the extensors of the hands and fingers. For this disorder we have found nothing so useful as the salino-sulphurous waters so common through the West, and especially in the state of Kentucky.

